# A Case of Strangulated Femoral Hernia during Pregnancy

**Published:** 1895-03

**Authors:** 


A CASE OF STRANGULATED FEMORAL HERNIA DURING PREGNANCY.—Gauctier (*Revue de Chirurgie*) reports a case of strangulated femoral hernia, on the right side, in a patient, aged forty, who had reached the ninth month of pregnancy. The hernia which had existed for six years, had previously given but little trouble, and the patient had never worn a truss. Kelotomy was performed twenty-four hours after the earliest symptoms of strangulation, and, after the reduction of a loop of deeply congested intestine, the sac was removed, and the opening closed by bringing Gimbernat's ligament into contact with the inner portion of Poupart's ligament, by means of oblique metallic sutures. The patient, who was delivered of a living and full-grown child, six days after the operation, made a speedy and complete recovery, and was discharged at the end of a month. The association of strangulated hernia with pregnancy must, the author states, be extremely rare, as he has been unable to find any previously recorded case; and, according to Berger, there is an incompatibility between these two conditions.—*British Medical ournal.*

